# Primary care, weather, and the missed opportunity for savings

**DOI:** 10.1093/haschl/qxag225

**Published:** 2026-08-27

**Authors:** Maranna Yoder, Nilesh Shinde, Guangqing Chi, Seth Freedman, Kosali Simon, Sonia Angell

**Affiliations:** O'Neill School of Public and Environmental Affairs, Indiana University, Bloomington, IN 47405, United States; O'Neill School of Public and Environmental Affairs, Indiana University, Bloomington, IN 47405, United States; Department of Geography, Indiana University, Bloomington, IN 47405, United States; O'Neill School of Public and Environmental Affairs, Indiana University, Bloomington, IN 47405, United States; O'Neill School of Public and Environmental Affairs, Indiana University, Bloomington, IN 47405, United States; Department of Epidemiology, Johns Hopkins University Bloomberg School of Public Health, Baltimore, MD 21205, United States

**Keywords:** health care delivery, weather, health economics, correlated shocks, value-based payment, system design

## Abstract

Extreme weather is a recurring source of stress on healthcare delivery, yet health systems remain largely reactive. Unlike an individual medical emergency, which the system absorbs one case at a time, a heat wave, wildfire smoke episode, or storm affects an entire population simultaneously. The resulting surge degrades care for every emergency patient, not only those the weather directly harmed. Primary care that anticipates weather-related risk and intervenes proactively can blunt that surge, but a central barrier to scaling such care is economic rather than clinical. Conventional prevention accounting counts only the benefit to the treated patient, and fee-for-service payment offers primary care practices only limited and poorly matched means to fund anticipatory care. Payment models that reward keeping patients healthy, such as global budgets, can address this, but only if risk adjustment accounts for environmental exposure so that providers serving vulnerable populations are not penalized. To address this, we outline 4 priorities: evaluations that capture system-wide costs; payment that rewards anticipatory care and adjusts for environmental risk; supply-side resilience in primary care, built jointly with public health agencies; and data infrastructure linking exposure to healthcare utilization.

Key takeaways

- Extreme weather events strain entire health systems at once, creating congestion costs that can harm many patients beyond those directly affected by the weather.
- Conventional economic evaluation and fee-for-service payment models fail to capture the value of preventing these system-wide disruptions, likely leaving anticipatory primary care undervalued and underfunded.
- Progress requires evaluations that capture system-wide costs of weather events; payment models that reward anticipatory care and clarify the division of labor between primary care and public health; building supply-side resilience; and data infrastructure linking exposure to utilization.

## A different kind of shock

When a patient presents to the emergency department (ED) with an acute myocardial infarction (a “heart attack”), the system is built to respond. Triage prioritizes care, cardiology is called, and care for others in the ED is preserved. However, when a heat wave sends a surge of patients to the same ED, capacity can be overwhelmed exactly when it is most needed. This is the difference between an *idiosyncratic* event, affecting one patient at a time, and a *correlated* shock, which strikes entire populations simultaneously.

A weather-driven surge degrades care for *all* emergency patients, not only those directly harmed by the event. When an ED is pushed beyond capacity, triage and attention to other urgent conditions is delayed, treatable pain and discomfort may be prolonged, and patients in psychiatric distress wait longer. Economists call this a *congestion externality*: a cost that spills onto patients beyond those triggered by the event. Temperature extremes alone contributed to over 69 000US deaths between 1999 and 2024, with medically fragile patients bearing disproportionate risk.^1,2^ In Mexico's largest healthcare subsystem, extreme heat raised ED visits by 6.9% and in-hospital deaths by 5.2%, with the mortality rise concentrated among patients admitted before the heat wave, isolating congestion from direct heat exposure.^3^ Similar dynamics appear during air pollution episodes in São Paulo and, outside the weather context, during the 2009 H1N1 pandemic.^4,5^ Direct US evidence on weather-driven hospital congestion remains limited, a gap we return to.

Because standard cost-effectiveness analysis only counts the benefit to the treated patient, it understates the value of preventing congestion. One proactive response is weather-responsive *primary care*, which anticipates a patient's weather-related risk and intervenes before the event, for example by screening older adults with comorbidities for air conditioning access. The formative barrier to this care is economic rather than clinical: without a payment and evaluation architecture that recognizes the value of prevention, it will remain difficult to scale and sustain.

## A taxonomy of weather shocks

Weather shocks can be classified along 2 dimensions relevant to system design: whether they generate a *demand surge*, a *supply disruption*, or both; and whether the disruption is *acute* or *sustained* (Table 1). Heat waves are distinctive because they do both, increasing patient demand while straining workforce and infrastructure. Hurricanes and winter storms disrupt supply by closing clinics, preventing staff travel, and destroying infrastructure. Wildfire smoke creates sustained demand surges across vast areas.^6^ Each shock calls for a different response: flexible capacity for demand surges, redundancy for supply disruptions, and coordination when both hit at once, which may be hardest where exposure is greatest and healthcare resources thinnest.^7^

**Table 1.** A taxonomy of weather shocks and weather-responsive primary care responses.

Shock type	Acute	Sustained	Matching primary care response
**Demand surge:** more patients seeking care	Heat wave	Wildfire smoke	*Flexible capacity.* Convert in-person primary care visits to telehealth; proactive outreach to high-risk patients.
**Supply disruption:** reduced capacity to deliver care	Winter storm, hurricane	Infrastructure damage	*Redundancy.* Mutual aid; distributed sites and primary care telehealth surge capacity; workforce contingency plans.
**Both:** demand rises as supply falls	Severe heat wave	Prolonged heat, grid strain	*Both, plus coordination.* Combine flexible capacity and redundancy with cross-provider coordination; the shortfall exceeds either shock alone.

Rows classify shocks by whether they raise demand, cut supply, or both; columns by whether the disruption is acute or sustained. Cells give representative shocks. The final column gives the weather-responsive primary care response each calls for.

## Payment architecture as the binding constraint

Even where the clinical case is strong, the payment architecture works against it. Under fee-for-service, some anticipatory outreach is technically billable: telephone evaluation and management, chronic care management, and principal care management codes can reimburse clinician time spent contacting patients. But reimbursement is modest, patient cost-sharing often applies, and the binding constraint is logistical as much as financial: a forecasted heat wave gives a practice days, not weeks, to reach hundreds of high-risk patients, a surge of non-visit work that fee-for-service revenue does not support staffing for in advance. Converting in-person visits to telehealth to reduce weather exposure often comes at reduced reimbursement. For the practice, prevention remains a cost burden rather than a funded strategy for keeping patients healthy.

Under value-based payment, including capitation, global budgets, or shared savings, organizations that keep patients healthy benefit from avoided ED visits and hospitalizations. The CMS AHEAD model, which provides hospital global budgets alongside enhanced payments to participating primary care providers, is especially relevant because it reaches safety-net providers serving the most weather-vulnerable populations. Hospital budgets are set prospectively from historical baseline revenue and adjusted for population characteristics and quality performance; they do not currently incorporate anticipated costs of extreme weather. Building forecast-based environmental exposure into budget-setting would prefund anticipatory care in high-risk areas and years, rather than after the fact. Because poorly managed chronic conditions raise weather vulnerability,^2^ chronic disease management itself reduces acute risk during extreme events, a return capturable under global budgets but invisible under fee-for-service.

A design concern follows. The most-affected populations are disproportionately low income and served by safety-net providers.^8^ If quality benchmarks and risk-adjustment formulas do not account for environmental exposure, value-based payment risks penalizing safety-net providers and widening gaps between high- and low-income areas. Moreover, cost-sharing may deter weather-vulnerable patients from seeking care during extreme events, so benchmarks built on observed utilization understate the true burden in high-cost-sharing plans.^8,9^

## Who should own anticipatory care?

A reasonable objection is that primary care, already stretched thin, cannot absorb another mandate, and that anticipatory care therefore belongs with public health agencies. The 2 are complements rather than substitutes. Public health agencies are better positioned for population-scale functions: issuing alerts, coordinating cooling centers, and mobilizing community health workers and resources. Primary care is better positioned for targeting highest-risk patients: adjusting medications that impair thermoregulation, managing chronic conditions that amplify weather risk, converting in-person to telehealth visits to maintain outpatient disease management during extreme weather, and identifying patients for early outreach. Population-based payment can finance this division of labor. A global budget gives health systems the flexibility and incentive to support and complement these primary care functions with contracts to local health departments and community-based organizations for outreach capacity. Anticipatory care can then draw on community-based infrastructure, expanding impact and decreasing demand on clinicians.

## Building the economic architecture

Four priorities follow. *First*, evaluations must account for congestion spillovers. Cost-effectiveness analyses should adopt a system perspective, comparing outcomes for all patients at affected facilities, not only weather-induced visits, so that averted congestion is counted as a benefit. Quasi-experimental designs comparing facility-wide outcomes across areas with and without anticipatory programs during the same weather events would provide needed estimates.

*Second*, supply-side resilience must be built into primary care. This means payment parity for telehealth during declared weather emergencies so that converting visits does not cost practices revenue; standing mutual-aid agreements that let practices share staff and triage lines across systems; and contingency staffing plans developed jointly with public health agencies rather than improvised event by event.

*Third*, payment models must reward anticipatory care. Risk adjustment should incorporate environmental exposure: AHEAD raises global budgets for hospitals serving higher-adversity populations using the Area Deprivation Index. Extending that logic to daily sub-county heat and air-quality exposure would keep providers in high-risk areas from being penalized. In the nearer term, existing chronic care management and principal care management codes could carry temporary weather-event modifiers that raise reimbursement and waive cost-sharing during forecasted events, funding outreach within fee-for-service while broader models mature.

*Fourth*, we need automated data infrastructure linking granular weather and air quality data to healthcare utilization at scale. All-payer claims and electronic health record databases, linked to daily sub-county exposures, are essential for estimating returns on weather-responsive care, for setting exposure-adjusted benchmarks, and for identifying patients whom outreach should reach first.

The clinical vision for weather-responsive healthcare is advancing. The technology for environmental monitoring and proactive outreach is evolving. What must change is the economic architecture holding back investment in primary care models that prevent and control weather-related risks at scale.

## Supplementary Material

qxag225_Supplementary_Data
